# Overlapping measures or constructs? An empirical study of the overlap between self-control, psychopathy, Machiavellianism and narcissism

**DOI:** 10.1016/j.fsisyn.2021.100141

**Published:** 2021-02-11

**Authors:** Matt DeLisi, Pedro Pechorro, João Maroco, Mário Simões

**Affiliations:** a Iowa State University, 203A East Hall, Ames, IA, 50011-1070, USA; b School of Psychology, University of Minho, Campus de Gualtar, 4710-057, Braga, Portugal; c William James Centre for Research. ISPA-Instituto Universitário, 1149-041, Lisboa, Portugal; d Faculty of Psychology, University of Coimbra, Rua Do Colégio Novo, 3000-115, Coimbra, Portugal

**Keywords:** Machiavellianism, Narcissism, Overlap, Psychopathy, Self-control

## Abstract

Overlap between self-control and dark triad traits (i.e., psychopathy, Machiavellianism, and narcissism) is potentially problematic for efforts to distinguish dimensions associated with elevated risk for antisociality and crime. The aim of the present study is to examine the potential overlap between self-control and psychopathy, Machiavellianism, and narcissism, with a focus on the Brief Self-Control Scale (BSCS) and the Dirty Dozen Dark Triad scale (DD). The sample consisted of 567 youth (*M* = 15.91 years, *SD* = 0.99 years, age range = 14–18 years) from Portugal. Exploratory and confirmatory factor analysis results from the pooled set of items of the BSCS and the DD measures revealed that both are valid and reliable measures of their respective constructs. However, consistent with previous research, the narcissism facet of the DD emerged as an independent factor. Our findings suggest that if such an eventual overlap is detected, it would be a question of problematic measures, not constructs.

## 1 Introduction

As largely independent constructs with their own literatures, self-control and psychopathy are indispensable risk factors for antisocial behavior. Across a variety of conceptual models in the social and behavioral sciences [[1], [2], [3], [4], [5]], self-control is a basic behavioral disposition characterized by behavioral disinhibition, sensation seeking, gratification delay, emotional regulation, and self-interest. Individuals who exhibit low self-control display a variety of deficits that relate to an inability or unwillingness to subordinate immediate desires to more prudent long-term solutions and tend to be self-centered, poorly tempered, impulsive, and poorly controlled. Indeed, low self-control is a consistently significant predictor of a broad swath of antisocial conduct [[6], [7], [8], [9], [10], [11], [12]] among diverse populations.

Across a variety of conceptual models in the social and behavioral sciences [[13], [14], [15], [16]] psychopathy is a personality disorder most similar to Antisocial Personality Disorder that presents a constellation of features that relate to core self-regulation problems, manipulative and exploitative interpersonal style, narcissism, impulsivity, sensation seeking, and global irresponsibility. Thus conceptually, self-control and psychopathy show a considerable amount of congruence. Spanning decades of research, numerous meta-analytic studies substantiate the empirical strength of both self-control [[17], [18], [19], [20]] and psychopathy [[21], [22], [23], [24]] as consistent and robust predictors of manifold forms of psychopathology and antisocial conduct.

A bourgeoning literature examined the relative contributions of self-control and psychopathy to antisocial behavior by including measures of both in the same statistical models. Most studies show that both self-control and psychopathy are significantly associated with various forms of delinquency and conduct problems [[25], [26], [27], [28], [29], [30]] although which of the two constructs is the better predictor has varied with some studies showing self-control is better [25,31], others showing psychopathy is better [[28], [31], [32]], and other studies showing the constructs are comparable [26,27,29,33].

An unresolved issue about the potential overlap between self-control and psychopathy pertains to measurement specifically if there is measurement overlap between the constructs. Wiebe [29] conducted exploratory factor analysis of 65 items taken from self-control and social control inventories and reported 12 factors that differentially relate to self-control and psychopathy. These factors were angry, antisocial cognition, attachment, diligent, guiltless, impulsive sociability, low commitment, manipulativeness, respect, risk seeking, shortsightedness, and sullen. Confirmatory factor analysis found that a two-factor structural model undergirds self-control and psychopathy. An antisociality factor defined by anger, low commitment, manipulativeness, antisocial cognition, and risk seeking and a self-direction factor defined by shortsightedness and diligence were the common threads to these conditions. Based on Wiebe’s [29] analyses, self-control and psychopathy are interrelated in that both conditions are instantiated by an antisociality disposition and an imprudent and hasty sense of self-direction.

In a recent comparative study, Armstrong et al. [34] examined self-control and psychopathy using exploratory and confirmatory factor analyses with the Grasmick et al. [35] self-control scale and Levenson et al. [36] self-report psychopathy scale. The Grasmick scale has six factors (impulsivity, simple tasks, risk seeing, physical, self-centered, and temper) meant to capture Gottfredson and Hirschi’s [2] criminological self-control construct. The Levenson scale has three factors (egocentricity, callousness, and antisocial) meant to capture the psychopathy construct. Exploratory factor analysis produced 11 factors including angry hostility, egocentricity, simple tasks, callousness, impulsivity, narcissism, physical activity, risk seeking, empathy, a miscellaneous category, and boredom. Confirmatory factor analyses produced eight factors including angry hostility, egocentricity, simple tasks, callousness, impulsivity, narcissism, physical activity, and empathy. Among these factors, five (angry hostility, simple tasks, callousness, impulsivity, and narcissism) contained items from both self-control and psychopathy scales. Physical activity contained only self-control items and empathy and egocentricity contained only psychopathy items. Armstrong et al. [34] thus show there is measurement contamination between self-control and psychopathy, which raises important questions whether similar findings would arise with different measures of self-control and psychopathy.

A related concept that traverses elements of self-control and psychopathy is the Dark Triad, which is a constellation of aversive personality features that includes the psychopathy, Machiavellianism, and narcissism facets of personality. Although these are theorized as distinct constructs, they collectively characterize individuals who have poor emotional and behavioral regulation, who have duplicitous and manipulative interpersonal styles, and who are self-centered and pursue their selfish interests with disregard for others. Several meta-analytic studies done so far indicate that the Dark Triad traits of personality are significantly related to assorted antisocial outcomes (e.g., aggression, victimization, violent, property, or substance offending) and/or personality pathology [[24], [38]]. Despite the fact Dark Triad traits and self-control are important constructs consistently associated with antisocial and criminal outcomes, research jointly examining them from a modern psychometric perspective is virtually non-existent to our knowledge.

### 1.1 Current study

With this in mind, we empirically examine potential overlap between self-control and psychopathy using different measures of both specifically the Brief Self-Control Scale and The Dirty Dozen which spans psychopathy, Machiavellianism and narcissism. We hypothesized that: a) Self-control as measured by the BSCS is significantly correlated with psychopathy, Machiavellianism, and narcissism as measured by the DD; b) Items from the BSCS and items from the DD load on their respective factors using exploratory factor analysis (EFA); and c) The different factors emerging from the EFA present a good fit in terms of confirmatory factor analysis (CFA).

## 2 Method

### 2.1 Participants

The sample examined in the present study was made up of 567 youth (*M* = 15.91 years, *SD* = 0.99 years, age range = 14–18 years), namely 256 females (*M* = 15.80 years, *SD* = 1.02, range = 14–18) and 311 males (*M* = 15.99 years, *SD* = 0.96, range = 14–18). No significant differences between genders were detected in terms of age (*F* = 3.38, *p* = .06), socioeconomic status (*U* = 38318.5, *p* = .41), or education (*F* = 0.63, *p* = .42). Most of the participants were Portuguese nationals (88.40%) with approximately nine years of education on average (*M* = 8.95, *SD* = 0.94).

### 2.2 Measures

*Brief Self-Control Scale* (BSCS; [39]. This is a brief 13-item self-report unidimensional measure of self-control. The BSCS includes items such as “I refuse things that are bad for me”; “I am able to work effectively toward long-term goals”; “Sometimes I can’t stop myself from doing something”. Items are rated on a 5-point Likert scale (ranging from 1 = *Not at all like me*, to 5 = *Very much like me*). The total score of the BSCS can be obtained by summing the items. The inverted items were reverse scored so higher scores reflect lower levels of self-control. The version of the BSCS validated in Portugal among the youth population was used in the current study [40]. The internal consistency for the current study estimated by Cronbach’s alpha (α) was very good (α = 0.93).

*Dirty Dozen* (DD; [41]. This is a brief 12-item tridimensional measure of the Dark Triad construct of personality composed of psychopathy, Machiavellianism, and narcissism. It is composed of four items for each trait that measure individual differences in psychopathy (e.g., “I tend to be unconcerned with the morality of my actions”), Machiavellianism (e.g., “I have used deceit or lied to get my way”), and narcissism (e.g., “I tend to seek prestige or status”). Items on the current study are rated on a 5-point ordinal Likert scale (ranging from 1 = *Not at all like me*, to 5 = *Very much like me*). The score of the three factors can be obtained by adding the respective items. Higher scores indicate higher levels of psychopathy, Machiavellianism, and narcissism. The version of the DD validated in Portugal among the youth population was used in the current study [[37], [40]]. The internal consistency as measured by Cronbach’s α ranged from good to excellent: Psychopathy α = .93, Machiavellianism α = 0.86, and Narcissism α = 0.88.

### 2.3 Procedures

The Ministry of Education (ME) of Portugal provided authorization to assess the participants of the present study. These participants came from public schools (i.e., State schools) of southern Portugal, that included the greater Lisbon, Alentejo and Algarve regions. This was a convenience sample not originally intended to be representative of the national student population, but it purposely contained male and female youth from urban (the city of Lisbon), and rural backgrounds (Alentejo and Algarve regions) to make it more diverse. Written parental authorization was previously obtained, and then the potential participants were themselves informed about the aims of our investigation and asked to collaborate voluntarily. Due to various motives some youth were excluded (e.g., those who could not read Portuguese, those who were reluctant to participate). The rate of participation was 89%. No form of compensation was given, including monetary compensation. The measures and sociodemographic questionnaire included in the present study were administered in small groups of participants.

### 2.4 Data analysis

IBM SPSS 27 [[52], [53]] software was used to conduct descriptive statistics analysis, correlational analysis (Pearson), group differences (Mann-Whitney’s *U* test, ANOVAs), reliability of measures, and exploratory factor analyses (EFA) with factor extraction using principal components Analysis (PCA) with varimax rotation. Pearson correlations were low if *r* < .20, high if *r* > 0.50, and moderate if in between. Cronbach’s alpha was considered very good if α > 0.90, good if α > 0.80, and adequate if α > 0.70 [[42], [51]].

EQS 6.4 [43] structural equation modeling (SEM) software was used to conduct the CFAs with Maximum Likelihood robust (MLR) methods and correlation covariance matrices. The usual fit indexes were used to evaluate model fit: Satorra-Bentler chi-square (SBχ^2^), degrees of freedom (df), Root Mean Square Error of Approximation (RMSEA), Akaike Information Criterion (AIC). Comparative Fit Index (CFI), and Incremental Fit Index (IFI). The >0.40 standardized loading cutoff was used to retain items [44]. Modification indices (MI) were used to improve the models when necessary. Items’ distributions were considered acceptable if they were not severely non-normal with absolute skewness and kurtosis values below 3 and 7, respectively [42].

The entire sample was used to examine the factor structures of the BSCS and DD with CFA (EQS 6.4; [43]. The sample was then randomly split in half, and EFA employing PCA with varimax rotation was used with the first half of the sample. Following that, the factor structure previously found was examined with CFA (EQS 6.4 [43]; using the second half of the sample [42]. No imputation for missing data was done because no missing values were detected.

## 3 Results

Table 1 presents the descriptive statistics of the BSCS and DD.

**Table 1:** Descriptive statistics of the BSCS and DD.

	*M* (*SD*)	Min-Max	Skewness	Kurtosis
BSCS	31.40 (7.76)	15–57	.47	-.28
DD-Psychopathy	7.60 (3.34)	4–17	1.08	.21
DD-Machiavellianism	8.19 (2.64)	4–15	.44	-.56
DD-Narcissism	11.66 (3.10)	4–19	-.03	-.79

*Note*. BSCS = Brief Self-Control Scale; DD = Dirty Dozen; M (SD) = Mean (Standard Deviation); Min-Max = Minimum-Maximum.

Table 2 shows the goodness-of-fit indexes of the BSCS and DD. The three factors of the DD were also examined individually as 1-factor models. The BSCS 1-factor model and the DD 3-factor model with modification indexes presented adequate fit. The DD-Psychopathy 1-factor model, the DD-Machiavellianism 1-factor model after correlating errors as suggested by modification indexes, and the DD-Narcissism 1-factor model with modification indexes also presented adequate fit.

**Table 2:** Goodness-of-fit indexes.

	SBχ^2^(df)	CFI	IFI	RMSEA	AIC
BSCS					
1-factor	274.98(65)	.99	.99	.07[.06-.08]	144.98
DD					
1-factor	1138.16(54)	.94	.94	.19[.18-.20]	1030.16
3-factor	303.72(51)	.98	.98	.09[.08-.10]	201.70
3-factor mi	221.23(49)	.99	.99	.08[.07-.09]	123.23
DD-Psychopathy					
1-factor	7.72(2)	.99	.99	.07[.02-.13]	3.72
DD-Machiavellianism					
1-factor	27.63(2)	.99	.99	.15[.10-.20]	23.63
1-factor mi	4.45(1)	.99	.99	.08[.02-.16]	2.45
DD-Narcissism					
1-factor	22.20(2)	.99	.99	.13[.09-.19]	18.20
1-factor mi	1.64(1)	.99	.99	.03[.00-.13]	-.35

*Note*. BSCS = Brief Self-Control Scale; DD = Dirty Dozen; mi = modification indexes.

Table 3 displays the Pearson correlation matrix of the measures used. The Psychopathy factor of the DD presented the highest correlation with the BSCS (Pearson *r* = .78, *p* < .001) followed by the Machiavellian factor (Pearson *r* = .68, *p* < .001) and the Narcissism factor (Pearson *r* = .45, *p* < .001). Intercorrelations for the DD factors ranged from 0.45 to 0.71.

**Table 3:** Pearson correlation matrix.

	BSCS	DD-Psychopathy	DD-Machiavellianism	DD-Narcissism
BSCS	1			
DD-Psychopathy	.78∗∗∗	1		
DD-Machiavellianism	.68∗∗∗	.71∗∗∗	1	
DD-Narcissism	.45∗∗∗	.54∗∗∗	.60∗∗∗	1

*Note*. BSCS = Brief Self-Control Scale; DD = Dirty Dozen.

∗∗∗*p* ≤ .001.

Next, EFA with PCA extraction and Varimax rotation was used to explore the structure that could emerge from the pooled set of items of the BSCS and the DD. The Kaiser–Myer–Olkin measure of sampling adequacy (KMO = 0.96) and Bartlett’s Test of Sphericity (χ^2^ = 5829.2, *p* ≤ .001) suggested the data was suitable for exploratory analysis. Results indicated the presence of three eigenvalues above 1 and a corresponding scree plot suggesting a three-factor solution accounting for 67.41% of the common variance. All the items loaded on their respective factors without significantly higher cross-loadings, except for items belonging to the Narcissism factor of the DD which loaded on a third separate factor. Table 4 presents the standardized loadings of the pooled set of items.

**Table 4:** Exploratory factor analysis standardized loadings.

	Factor 1	Factor 2	Factor 3
Brief Self-Control Scale			
I have a hard time breaking bad habits.	**.58**	.30	.21
I am lazy.	**.57**	.18	.11
I say inappropriate things.	**.67**	.24	.34
I do certain things that are bad for me, if they are fun.	**.70**	.33	.25
I refuse things that are bad for me.	**.71**	.39	.15
I wish I had more self-discipline.	**.74**	.22	-.08
I am good at resisting temptation.	**.72**	.30	.23
People would say that I have iron self-discipline.	**.80**	.18	-.02
Pleasure and fun sometimes keep me from getting […].	**.69**	.26	.22
I have trouble concentrating.	**.74**	.27	.07
I am able to work effectively toward long-term goals.	**.77**	.34	.02
Sometimes I can’t stop myself from doing something […].	**.72**	*.44*	.27
I often act without thinking through all the alternatives.	**.75**	.30	.12
Dirty Dozen			
I tend to lack remorse. (P)	.35	.**65**	.25
I tend to be unconcerned with the morality of my actions. (P)	.32	**.79**	.15
I tend to be callous or insensitive. (P)	.29	**.72**	.14
I tend to be cynical. (P)	.32	**.71**	.30
I tend to manipulate others to get my way. (M)	*.42*	**.59**	.25
I have used deceit or lied to get my way. (M)	*.49*	**.59**	.21
I have used flattery to get my way. (M)	*.46*	**.58**	.31
I tend to exploit others towards my own end. (M)	*.46*	**.59**	.36
I tend to want others to admire me. (N)	.09	.16	**.90**
I tend to want others to pay attention to me. (N)	.06	.16	**.90**
I tend to seek prestige or status. (N)	.16	.28	**.81**
I tend to expect special favors from others. (N)	.30	*.54*	**.60**

*Note*. Cross-loadings in italics. P = Psychopathy, M = Machiavellianism, N = Narcissism.

Finally, CFA was used to confirm the 3-factor model that emerged from the EFA. This 3-factor model (SBχ^2^ = 682.46; df = 272; CFI = 0.99; IFI = 0.99; RMSEA = 0.07[0.06-0.08]; AIC = 138.46) revealed a good adjustment in terms of goodness-of-fit indexes without using modification indexes when compared to a 1-factor model (SBχ^2^ = 525.02; df = 54; CFI = 0.96; IFI = 0.96; RMSEA = 0.18[0.16-0.19]; AIC = 417.02). Table 5 presents the CFA standardized loadings of the pooled set of items.

**Table 5:** Confirmatory factor analysis standardized loadings.

	Factor 1	Factor 2	Factor 3
Brief Self-Control Scale			
I have a hard time breaking bad habits.	.69		
I am lazy.	.65		
I say inappropriate things.	.75		
I do certain things that are bad for me, if they are fun.	.80		
I refuse things that are bad for me.	.84		
I wish I had more self-discipline.	.72		
I am good at resisting temptation.	.80		
People would say that I have iron self-discipline.	.74		
Pleasure and fun sometimes keep me from getting […].	.75		
I have trouble concentrating.	.76		
I am able to work effectively toward long-term goals.	.82		
Sometimes I can’t stop myself from doing something […].	.89		
I often act without thinking through all the alternatives.	.80		
Dirty Dozen			
I tend to lack remorse. (P)		.76	
I tend to be unconcerned with the morality of my actions. (P)		.78	
I tend to be callous or insensitive. (P)		.69	
I tend to be cynical. (P)		.82	
I tend to manipulate others to get my way. (M)		.88	
I have used deceit or lied to get my way. (M)		.87	
I have used flattery to get my way. (M)		.92	
I tend to exploit others towards my own end. (M)		.90	
I tend to want others to admire me. (N)			.83
I tend to want others to pay attention to me. (N)			.82
I tend to seek prestige or status. (N)			.84
I tend to expect special favors from others. (N)			.83

*Note*. P = Psychopathy, M = Machiavellianism, N = Narcissism.

## 4 Discussion

Comparative or “head-to-head” tests between self-control and psychopathy in criminology produced a wide range of findings about the relative strength and specificity of these constructs as general theories of crime. Due to the clear conceptual overlap between self-control and psychopathy, an important next step is to perform factor analytic studies of measures of these constructs to assess whether they overlap. Recently, Armstrong et al. [34] reported considerable overlap in the constructs using the Grasmick et al. self-control scale and Levenson et al. psychopathy scale. Indeed, Armstrong et al. [34, p. 8] advised, “With regard to the measurement of self-control and psychopathy, overlap between the GSCS and LSRP may be regarded as a conceptual issue coming home to roost as a methodological problem. Original specifications of psychopathy included a wide range of dispositions that were thought to underlie the psychopathic personality type. Similarly, Gottfredson and Hirschi’s enumeration of the elements of self-control ranges quite wide, perhaps as a result of the authors efforts to reconcile incongruent lines of research with the background assumptions of control theory. Nonetheless, the LSRPS and the GSCS are faithful to the conceptualizations of the constructs that they purport to measure. Therefore, our results suggest that rather than giving attention to the refinement of these measures, work directed at furthering our understanding of the role of individual differences in the explanation of crime should devote attention to identifying and measuring the distinct traits that explain between individual differences in the tendency to commit crime.” We followed their advice in the current study.

Unlike Armstrong et al. [34], we found no evidence of overlap using the BSCS and DD suggesting that self-control and psychopathy are distinct as constructs and as measures of those constructs. What explains these contrasting findings? Consistent with Armstrong et al. we suspect that a limitation of the Grasmick scale is that it too earnestly attempted to operationalize a theory of self-control [2] that because it was touted as a general theory was too expansive and ambitious. As such, the Grasmick scale attempts to cover wide terrain some of which (e.g., the physical activity dimension) is not as essential to self-regulation as impulsivity. In contrast, the BSCS is a parsimonious measure that narrowly encompasses attentional control and many facets of Conscientiousness, and is not beholden so to speak to a general theory. A related issue is that the BSCS is unidimensional whereas the Grasmick scale has several dimensions or latent factors, which makes it more difficult to replicate its factor structure. An obvious next step is to replicate the current study with additional measures of self-control and psychopathy.

The current study was limited in a variety of ways. Although the DD shows adequate validity and reliability, its brevity of items comes at the expense of omitting important features of psychopathy, such as behavioral disinhibition and interpersonal antagonism (see, Miller et al. [45]). It is possible that a more comprehensive measure of psychopathy that was not *also* measuring narcissism and Machiavellianism would produce different results. Moreover, Machiavellianism and psychopathy loaded on the same factor in both the exploratory and confirmatory factors analyses, which suggests a certain amount of redundancy between these constructs as suggested by prior meta-analytic research (see, Muris et al. [24]). By definition, the interpersonal features of psychopathy involve a calculating, cunning, and manipulative style thus a highly psychopathic person is *ipso facto* also Machiavellian. Additional studies with data from diverse populations can help address the redundancy issue. An inherent limitation of school samples such as the current study is these adolescents are normative in their personality and behavioral functioning [46] and do not evince clinical deficits in self-control or pronounced psychopathic features. As such, another important avenue for future research is to replicate our work with adjudicated or correctional samples shown to display much greater variance and extreme scores on low self-control and high psychopathy [31,40,[47], [48], [49], [50]].

## 5 Conclusion

In closing, the past few years contained a flurry of studies that directly compare self-control and psychopathy as general or unified theories of antisocial behavior, and to date there are contrasting findings. Although it is clear that both constructs are robust predictors of antisocial behavior, the relative value of them, and the independence of measures of these constructs are still open empirical questions. Our findings suggest that if such an eventual overlap is detected, it would be a question of problematic measures, not constructs.

## Funding

This study was partially conducted at the Psychology Research Centre (CIPsi/UM PSI/01662) School of Psychology, 10.13039/501100008814University of Minho, and was partially funded by the 10.13039/501100001871Portuguese Foundation for Science and Technology (FCT) and the Portuguese Ministry of Science, Technology and Higher Education (UID/PSI/01662/2019) through the Portuguese State budget (UIDB/01662/2020).

## Declaration of competing interest

The authors declare that they have no known competing financial interests or personal relationships that could have appeared to influence the work reported in this paper.
